# Citotoxicity evaluation of three dental adhesives on vero cells *in vitro*

**DOI:** 10.4317/jced.53039

**Published:** 2017-01-01

**Authors:** Raisa-Queiroz Catunda, Jeymesson-Raphael-Cardoso Vieira, Erwelly-Barros de Oliveira, Eliete-Cavalcanti da Silva, Veruska-Lima-Moura Brasil, Danyel-Elias-da Cruz Perez

**Affiliations:** 1 MSc student, Department of Clinical and Preventive Dentistry, Federal University of Pernambuco, Recife, Pernambuco, Brazil; 2 Professor, Department of Histology and Embryology, Federal University of Pernambuco, Recife, Pernambuco, Brazil; 3 Undergraduate, Department of Histology and Embryology, Federal University of Pernambuco, Recife, Pernambuco, Brazil; 4 PhD student, Department of Clinical and Preventive Dentistry, Federal University of Pernambuco, Recife, Pernambuco, Brazil; 5 Professor, Department of Clinical and Preventive Dentistry, Federal University of Pernambuco, Recife, Pernambuco, Brazil

## Abstract

**Background:**

To evaluate, *in vitro*, the potential cytotoxicity of three different dental adhesives systems (Adper Single Bond 2 -SB, Silorane System Adhesive Bond -SSAB and Single Bond Universal -SBU) on cultivated Vero cells after different contact times.

**Material and Methods:**

The cells were cultured in a concentration of 2 x 105 cells/mL for 24h and grown to sub-confluent monolayers. VERO cells were exposed to 25µl of conditioned extracts obtained from 24h, 48h and 72h immersion of adhesive samples in culture medium (DMEM), immediately after polymerization. Fresh DMEM was used as negative control. Cell metabolism was evaluated by the MTT assay (3-(4,5-dimethylthiazol-2-yl)-2, 5diphenyl-tetrazolium bromide). The data were analyzed statistically by ANOVA, considering a significance of 5%.

**Results:**

The values of cell viability ranged from 94.2% at 72h (SBU) to 109.6% at 48h (SB). The mean percentage of viability after exposure to the extracts of SB, SSAB and SBU were 103.2%, 100.63% and 97.43%, respectively. There was no statistically significant difference (*p*= 0.342) between the experimental and negative control groups.

**Conclusions:**

At all exposure times, all adhesives tested in this study presented no cytotoxicity to Vero cells *in vitro*.

** Key words:**Biocompatibility, cytotoxicity, dental adhesives, Vero cells.

## Introduction

As biomaterials, dental adhesive systems must satisfy the requirement of biocompatibility, which can be defined as the ability of materials to perform their specific functions when applied on a living tissue of a particular host, without causing damage or injury (1-4).

The assessment of cytotoxicity is a prerequisite for the biocompatibility evaluation of the materials (1-4). By definition, the cyto-toxicity of an agent means toxicological risks by a material or its extract in cell culture (3,5). The interaction of the materials and its components with the cells at the molecular level is responsible for many of the immune alterations and genotoxicity registered (6), as well as tissue reactions such as inflammation and necrosis (7).

The assessment of cytotoxicity of dental adhesives is indispensable because of the close contact with vital dentin and pulp tissue, and in case of accidents involving other surrounding tissues such as epithelium or connective tissue of the oral mucosa (4). Although widely used in clinical dentistry practice without reports of significant adverse effects on pulp tissue, the individual components of adhesive systems proved to be cytotoxic to various cells, such as pulp and gingival cells (8,9). Many of the substances released show diverse degrees of cytotoxic activity *in vitro* (10). In addition, cellular response varies according to the methodology tested (11), demonstrating the need to test each one.

Dental adhesives considered as golden standard in Dentistry are known as total-etching systems or 5th generation systems. Self-etching systems are the ones that do not use a separated step for enamel/dentin etching, they have a self-etching primer/two bottle system (6th generation) or a self-etching adhesive/one bottle system (7th generation) (12).

However, little is known regarding cytotoxicity of these new adhesive systems, mainly Single Bond Universal (3,13-15). Therefore, it is necessary to assess the degree of cellular damage caused by these new generation agents. The purpose of this study was to evaluate the response of cultured Vero cells to three different dentin adhesives on different times of exposure by observing cell metabolic activity using the methyltetrazolium (MTT) assay.

## Material and Methods

-Materials

Three commercial dental adhesives ( Table 1) with different clinical application procedures (total etching and self-etching systems) were evaluated: Adper SingleBond 2 (3M ESPE, Saint Paul, USA), Silorane System Adhesive Bond (3M ESPE, Saint Paul, USA) and Single Bond Universal (3M ESPE, Saint Paul, USA).

**Table 1:**
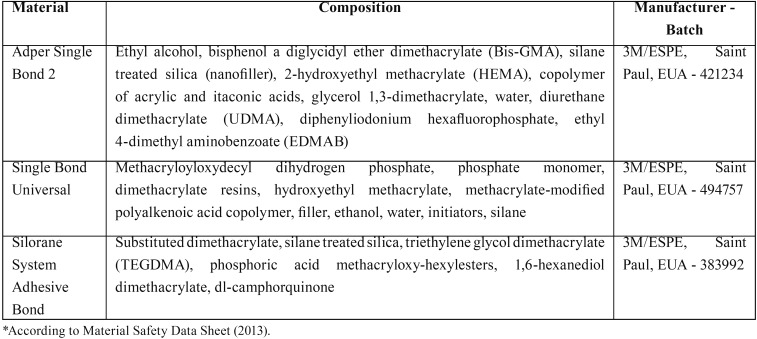
Materials used in this study*.

-Cell Culture

VERO cell line (CCL-81, Rio de Janeiro, Brazil) was obtained from Cell Culture Laboratory, Department of Histology and Embriology, Federal University of Pernambuco, Recife, Brazil. The cells were maintained at 37°C in a humidified 5% CO2 atmosphere. Growth monitoring cell was performed by using an inverted microscope (Eclipse TS 100, Nikon, Japan). The culture medium used was Dulbecco’s Modified Eagle Medium (DMEM, Sigma Chemical Co., Saint Louis, USA) supplemented with 10% fetal bovine serum (Cultilab Ltda, Campinas, Brazil) and 1% antibiotic-antimycotic solution (10,000 UI of penicillin, 10 mg of streptomycin in 0.9% sodium chloride; Sigma Chemical Co., Saint Louis, USA). Cultures were supplied with fresh medium every 3 days until an adequate number of cells was obtained. The cells were counted in a Neubauer chamber after 1:10 dilution in Try-pan Blue Dye (10µL of cells in 90µL of Trypan Blue). The cells (2x105 cells/ mL of DMEM per well) were transferred to the culture plate (Sigma-Aldrich, Munich, Germany) and incubated for 24h at 37°C in 5% CO2 and 95% air to stabilize the cells.

-Samples preparation

All samples were made according to the International Organization for Standardization (10993-12), part 12: Sample preparation and reference materials, 2012 (16).

Ninety samples of the adhesives systems were obtained by dripping adhesive on a cylindrical matrix of orthodontic elastics (5mm diameter x 2 mm height) placed on a strip of polyester and light-cured for 20 seconds with a halogen light source (Optilight Plus, Gnatus, São Paulo, Brazil) at a power density of 500 mW/cm2. The halogen light source was calibrated with a radiometer (Deme-tron, Kerr Corp., CT, USA) to every 5 samples made. To ensure aseptic conditions, the discs were prepared in a laminar flow chamber (VECO, São Paulo, Brazil). Immediately after the cure, the samples were removed from the cylindrical matrix. The samples thickness was measured in two areas using a digital caliper with an accuracy of 0.01 mm. All specimens were prepared by the same operator. The samples were exposed to ultraviolet light for 45 minutes to prevent bacterial contamination.

-Extracts and experimental groups

Extracts were prepared by soaking samples in DMEM that was stored in Falcon tubes. The samples had a mean size of 0.5 cm2, which is within the recommended range of 0.5-6.0 cm2/mL suggested by the International Organization for Standardization (2012) (16). In order to conditioning the medium, the specimens were immersed in DMEM for 24 h, 48h or 72h ( Table 2). After the period of conditioning, the discs were removed and conditioned DMEM filtered (Syringe filter 0.22µm, TPP, Darmstadt, Germany) to eliminate solid particles. The amount of growing medium required for each specimen was 1.414 mL.

**Table 2:**
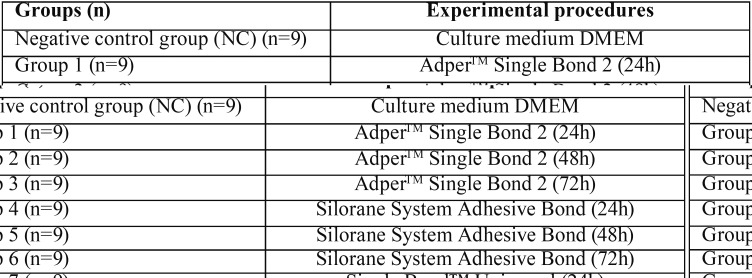
Sample groups and experimental procedures.

-Cytotoxicity assay

**Figure 1:**
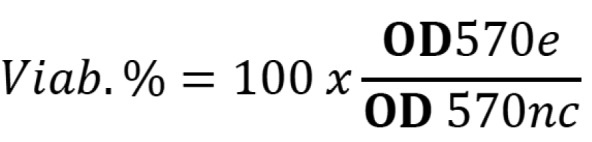
Equation.

In 96-well culture plates (TPP, Darmstadt, Germany), 2×105 cells in 1mL of DMEM per well were cultured and grown to sub-confluent monolayers for 24h. The culture medium was then replaced with equal volumes (25µl) of adhesive extracts (conditioning medium), using the culture medium itself as negative control. The evaluation of the cytotoxic activity was determined by the colorimetric method bromide (3 - {4,5-dimethylthiazol-2-yl} -2,5-diphenyl tetrazolium bromide) (MTT). After 24 hours incubation, 25 µl (5 mg/ml) of MTT solution was added to each well and the plates were incubated for 3 hours. The MTT was then removed and 25µl per well dimethyl sulphoxide (DMSO) was added to each well to dissolve the formazan crystals. According to ISO 10993-12 (14), a decrease in the number of living cells results in a decrease in the metabolic activity in the sample. Such decrease directly correlates with the amount of blue-violet formazan formed, as monitored by the optical density (OD) at 570 nm. To calculate the reduction of viability compared to the negative control, the following equation is used, (Fig. 1):

OD570e is the mean value of the measured optical density of the 100% extracts of the test sample and OD570nc is the mean value of the measured optical density of the negative control. The lower Viab.% value means higher the cytotoxic potential.

The cell viability was classified in non-cytotoxic (more than 90 per cent cell viability), slightly cytotoxic (60-90 per cent cell viability), moderately cytotoxic (30-59 per cent cell viability) and severely cytotoxic (less than 30 per cent cell viability), according to Ahrari *et al.* (17).

-Statistical analysis

Data were compared by ANOVA for two interactional factors (time of exposure and adhesives; adhesives and negative control group) and had a 5% margin of error.

## Results

**Figure 2:**
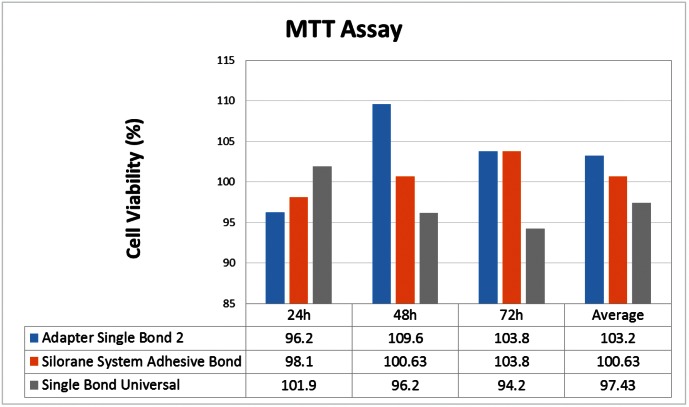
Effect of the dental adhesives on Vero cells after 24, 48 and 72 hours of exposure. Data are expressed as a percentage of the negative control cultures (*p*= 0.342).

The results of cell viability (MTT assay) showed that all bonding agents had no cytotoxic effects (all values >90%) and the values of cell viability ranged from 94.2% (Group 9) to 109.6% (Group 2) (Fig. 2). The mean percentage of viability after exposure to the extracts of Single Bond (SB), Silorane System Adhesive Bond (SSAB) and Single Bond Universal (SBU) were 103.2%, 100.63% and 97.43%, respectively (Fig. 2). No statistically significant difference was observed (*p*=0.342) between the experimental and negative control groups.

**Figure 3:**
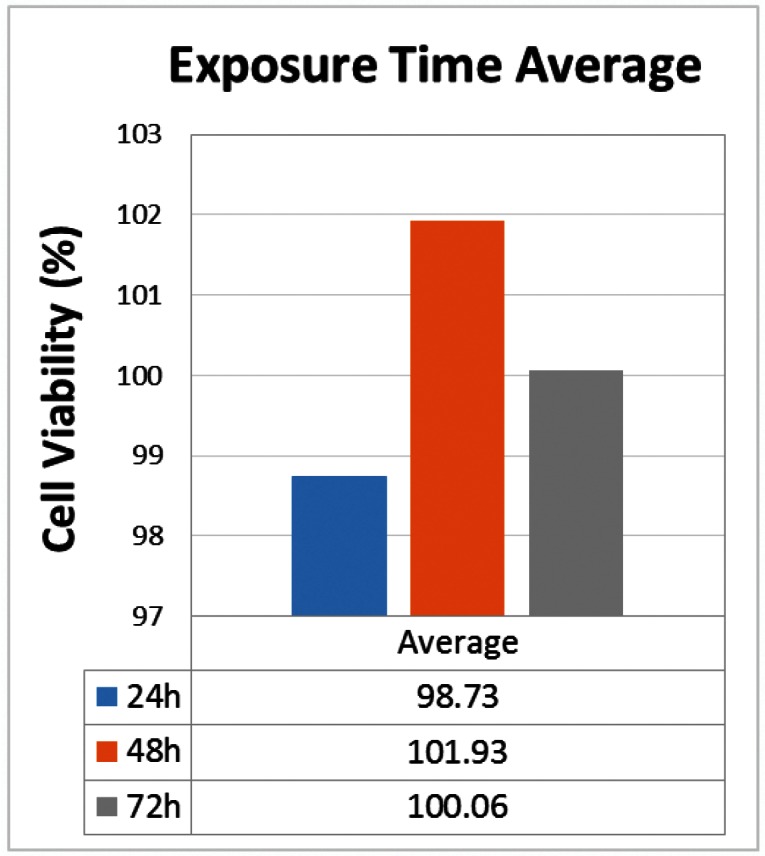
Mean cell viability (%) according to three different exposure times (*p*=0.724).

Considering the average of viable cells in all tested materials the lowest score was observed after 24h (98.73%) of adhesive exposure to the culture medium (Fig. 3), although no statistically significant difference was observed (*p*=0.724).

## Discussion

Oral cells may be exposed to polymers used in Dentistry, as the ones in dental adhesives, when they been exposed to gingival tissue, or indirectly when products are released from the polymers and migrate towards the pulpal or surrounding tissues (15). *In vivo* studies provide the most authoritative results on biocompatibility. However, for ethical reasons, cytotoxicity evaluation of materials is mostly carried out on cell cultures (15,16). The present study was conducted on Vero cells, according to ISO 10993- 5 (2009) recommendations (18). These cell lines have well-defined culturing characteristics in experimental settings and have been previously used for this purpose (17).

Nowadays, two different bonding strategies are well accepted to obtain physical interaction between resin and dentine (19). The first one is known as etch-and-rinse. It is based on the total removal of the smear layer and demineralization of the subjacent dentine, and is considered as golden standard for predictable adhesion to the tooth. With three steps adhesives (fourth generation), the etching is performed with phosphoric acid (30-40%) during 15 seconds. After the phosphoric acid removal, the primer and adhesive may be used. It can also be done by the two steps technique (fifth generation), which involves the use of the phosphoric acid and then the adhesive and primer at once (12). The second etching strategy (sixth and seventh generations) is based on using acid monomers, which are called self-etching primers or self-etching adhesives. They demineralize partially or completely the smear layer and subjacent dentine, incorporating and using them as a substratum for the adhesion (20).

The self-etching systems have acid hydrophilic monomers, HEMA (2-Hydroxyethyl- methacrylate) and dimethacrylate bifunctional. The increase in the concentration of acid monomers is needed to dissolve the smear layer and etch the subjacent dentine, and water is used as a mean of ionization of these acids resinous components. HEMA is added as a solvent, because some of the acidic monomers are not soluble in water directly (21).

The tested bonding agents can be classified, based on their adhesion strategy, into various generations and systems. Adapter Single Bond (fifth generation or three bottle system), Silorane System Adhesive Bond (sixth generation or two bottle system) and Single Bond Universal (seventh generation or one bottle system), which etch and prime simultaneously, because they have a high concentration of acidic resin monomers. The first two agents require the use of phosphoric acid or a primer containing methacrylate-based monomers as well. There are few *in vitro* studies which compare 5th generation adhesives with new generations, and the comparison is usually made based on the etching technique. Most results showed that 6th and 7th generations are more cytotoxic than 5th generation adhesives (13-15). However, in the current study, no cytotoxic effects were observed in the adhesives evaluated.

Any type of dental material needs to be qualified for clinical use. Thus, these materials should be tested for their effectiveness and biocompatibility. There are many different *in vitro* studies that may be used for this purpose. MTT is a well-known and accepted cytotoxicity assay (4-7,18). Viable cells reduce the MTT tetrazolium salt to a blue and insoluble product formazan, which precipitates in the cytoplasm. The advantages of this assay are accuracy, speed and no need to use radioisotope (18).

In this study, the choice of test materials from the same manufacturer intended to evaluate the possible cytotoxicity based on the different adhesives generations and systems. As the three chosen types are highly used in clinical practice worldwide, it is important that the evolution of materials and techniques are also reinforced by scientific tests. The extracts used in the present study were obtained by immersing the samples in the culture medium for either 24, 48h or 72 h, which is a sufficient period for 85% of the unreacted monomer to be released (19).

The current study showed all of the tested adhesives presented a very high percentage of viable cells (>90%). Mean exposure time (mean of all adhesives tested) showed a slightly increase in the cell viability from 24h (98.73%) to 48h (101.93%) and a slight decrease at 72h (100.06%). The individual mean of SB (103.2%) and SSAB (100.63%) were above the negative control (100%). These results are important and reinforce that the adhesives tested were not cytotoxic in this assay. Although the components (HEMA, TEGDMA, phosphate monomer) might seem toxic after the initial monomer release (22), they did not interfere in cell growth, which may have led them to proliferate as they usually would in favorable conditions. Similar findings have been observed in previous studies using Easy Bond, with percentages of cell viability from 103.37% to 110.39%, depending on the methodology (23,24). Cell viability over 100% observed in some experimental groups can be attributed to the accumulation of cell waste products and metabolites in the control negative cells, as the media had not been changed for the last 48h whilst our cells seemed almost 100% confluent at 24h. Thus, the programmed cell death took place to rescue some cells on the expense of others (23).

In this experiment, SB showed no citotoxicity, differing from recent study (3), which found values that ranged from 33% to 51%. Another study (29) found values that ranged from 64.56% to 82.33%. In the last study (25), the authors used unpolymerized liquid form of adhesives directly on cells, decreasing the cell viability when compared with the current study. We followed ISO (2012) (16) recommendations for sample preparations, which considers as appropriate pieces of approximately 10 mm x 50 mm or 5 mm x 25 mm to calculate and obtain a proper quantity of medium. Additionally, the mentioned studies (3,25) used different type of cell lines, the fibroblast cell line (L929), and mouse fibroblasts cells (3T3) and bovine dental papilla-derived cells (SV3NeoB), respectively.

Although no statistically significant difference was found (*p*=0.342), Group 9, a new one-step self-etching adhesive, showed a slight decrease (94,2%) in Vero cells than the other fifth (103,8%) and sixth generation (103,8%) adhesives, which was similarly observed in previous study involving SB and self-etching adhesives (13). In accordance with recent studies, although no cytotoxic effects have been observed, the findings of SUB can be attributed to one of its components, the phosphate monomer. Amongst seven different solvents, it has been considered the most cytotoxic followed by HEMA, THF, acetone and ethanol (22).

Group 1 had 96.2% of cell viability, followed by Group 4 (98.1%) and Group 7 (101.9%). The average of all tested materials showed the lowest score after 24h (98.73%) of adhesive exposure to the culture medium. This can be explained because just 50-75% of the monomers polymerize, the rest remain as free radicals, mainly reactive oxygen species (ROS), which have the ability to diffuse constituting a biological risk to cells at immediate times of exposure (26). Group 2 had 109.6% of cell viability, followed by Group 5 (100,63%) and Group 8 (96.2%). This finding can suggest a little cell growth in Group 2 and 5 and a slight decrease in Group 8 (20,26). In addition, Group 3 and Group 6 showed the same percentage of cell viability (103.8%).

In summary, the differential viability/cell proliferation induced by the materials tested could be attributed to the different components, the interactions between them, the degree of resin polymerization and the type of cultured cell. According to this study, all adhesives showed no cytotoxicity. However, further *in vitro* and *in vivo* studies should be performed in order to find more accurate results, as in clinical practice there are other non-reproduced variants *in vitro*.
